# A comprehensive map of microbial biomarkers along the gastrointestinal tract for celiac disease patients

**DOI:** 10.3389/fmicb.2022.956119

**Published:** 2022-09-13

**Authors:** Juliana Estefanía Arcila-Galvis, Viviana Loria-Kohen, Ana Ramírez de Molina, Enrique Carrillo de Santa Pau, Laura Judith Marcos-Zambrano

**Affiliations:** ^1^Computational Biology Group, Precision Nutrition, and Cancer Research Program, IMDEA Food Institute, Madrid, Spain; ^2^Computational Epigenomics Laboratory, Faculty of Medical Sciences, Newcastle University, Newcastle, United Kingdom; ^3^Nutrition and Clinical Trials Unit, GENYAL Platform IMDEA-Food Institute, Madrid, Spain; ^4^Departamento de Nutrición y Ciencia de los Alimentos, Faculty of Pharmacy, Universidad Complutense de Madrid, Madrid, Spain

**Keywords:** microbiota, celiac disease, 16S rRNA gene, gluten free diet (FGD), metataxonomy

## Abstract

Dysbiosis of the microbiome has been related to Celiac disease (CeD) progress, an autoimmune disease characterized by gluten intolerance developed in genetically susceptible individuals under certain environmental factors. The microbiome contributes to CeD pathophysiology, modulating the immune response by the action of short-chain fatty acids (SCFA), affecting gut barrier integrity allowing the entrance of gluten-derived proteins, and degrading immunogenic peptides of gluten through endoprolyl peptidase enzymes. Despite the evidence suggesting the implication of gut microbiome over CeD pathogenesis, there is no consensus about the specific microbial changes observed in this pathology. Here, we compiled the largest dataset of 16S prokaryotic ribosomal RNA gene high-throughput sequencing for consensus profiling. We present for the first time an integrative analysis of metataxonomic data from patients with CeD, including samples from different body sites (saliva, pharynx, duodenum, and stool). We found the presence of coordinated changes through the gastrointestinal tract (GIT) characterized by an increase in *Actinobacteria* species in the upper GIT (pharynx and duodenum) and an increase in *Proteobacteria* in the lower GIT (duodenum and stool), as well as site-specific changes evidencing a dysbiosis in patients with CeD’ microbiota. Moreover, we described the effect of adherence to a gluten-free diet (GFD) evidenced by an increase in beneficial bacteria and a decrease in some *Betaproteobacteriales* but not fully restoring CeD-related dysbiosis. Finally, we built a Random Forest model to classify patients based on the lower GIT composition achieving good performance.

## Introduction

Celiac disease (CeD) is an autoimmune disease affecting the small intestine, ranging from intraepithelial lymphocytosis to the total atrophy of intestinal villi as a response to gluten consumption (Dieli-Crimi et al., 2015). This disease is characterized by a genetic predisposition given by the alleles coding for the Human Leukocyte Antigens (HLA) HLA-DQ2 and/or HLA-DQ8, the presence of antibodies against transglutaminase type 2 (TG2), and immunoglobulins IgA and IgG anti-gluten as well as gastrointestinal symptoms when consuming gluten-containing foods (Dieli-Crimi et al., 2015). The HLA variant with a higher association with CeD is HLA-DQ2, present in more than 90% of patients; approximately half of the remaining patients possess an HLA allele coding for HLA-DQ8 (García-Santisteban et al., 2021).

Besides the implication of HLA genes in CeD, more than 40 loci outside of the HLA region have been linked with the disease (Gnodi et al., 2022). The risk effect of these non-HLA variants is estimated to account for 15% of the disease’s genetic component (Withoff et al., 2016). All of the genetic variants discovered to date, including both HLA and non-HLA Single Nucleotide Polymorphisms (SNPs), explain more than half of the heritability (García-Santisteban et al., 2021). It should also be noted that only 5% of the identified SNPs were found in coding regions, with the remaining 5 and 9% found in 5’ and 3’ untranslated regions, respectively, meaning that 81% of the SNPs found were in intergenic or intronic regions, implying that their function could be to regulate gene expression, possibly through interactions with transcription factors or proteins able to regulate chromatin status, that is, epigenetic modifications, in this sense CeD-related epigenetic changes have been described, including DNA methylations, histone modifications, and non-coding RNA expression (Gnodi et al., 2022). However, current evidence suggests that other non-gluten environmental factors can influence disease risk in addition to genetic aspects (Sollid and Lundin, 2020).

One of the non-gluten environmental factors influencing the development of CeD is the gut microbiome, the complex of microorganisms that reside in the gut and participate in the body’s metabolic, physiological, and immune processes (Cenit et al., 2016; Levy et al., 2017; Chander et al., 2018; Krishnareddy, 2019). Studies examining the microbiome in CeD suggest that the intestinal microbiome of those with the condition is altered, with opportunistic bacteria clades increasing while beneficial clades decreasing, resulting in a condition known as dysbiosis (Belkaid and Hand, 2014).

Dysbiosis could lead to an alteration in the intestinal barrier. Sustained inflammation or infection (overgrowth of pathogenic bacterial clades) can lead to deregulation in the expression of adhesion molecules at tight junctions leading to the entry of microbes and toxic substances facilitating the entry of incompletely digested gliadin peptides—the gluten-derived protein that triggers proinflammatory cytokines in CeD—in lamina propria (Belkaid and Hand, 2014; Chander et al., 2018; Valitutti et al., 2019). CeD causes a change in the architecture of the small intestine; focal epithelial barrier defects occur with increased apoptosis and altered tight junction–mediated permeability resulting in a loss of ions and water to the gut lumen, particularly barrier-forming claudins (claudin-3, claudin-5, and claudin-7) are down-regulated while the channel-forming claudins (claudin-2 and claudin-15) are up-regulated, which increases the selective paracellular solute transport (Schumann et al., 2017). Additionally, the disassembly of zonulin, a protein that reversibly regulates intestinal permeability by modulating intercellular tight junction molecules, has been associated with the disease (Lammers et al., 2008). Zonulin is neither specific nor unique to CeD, as other proinflammatory mediators of barrier and tight junction down-regulation, such as tumor necrosis factor A and interferon-g, have been described in active CeD (Schumann et al., 2017). However, gluten peptides and some enteric bacteria, such as *Escherichia coli*, can induce this protein, suggesting an implication in CeD pathogenesis (De Palma et al., 2010).

Besides zonulin disassembly, gliadin peptides and gut dysbiosis can similarly activate innate and adaptive immune systems (Chibbar and Dieleman, 2019). Gram-negative bacteria trigger the innate immune system by activating Toll-like receptors (TLR-4), and CD14 complexes recognize bacterial endotoxins and lipopolysaccharide, prompting the innate immune system to release proinflammatory cytokines (Chibbar and Dieleman, 2019; Valitutti et al., 2019). In patients with CeD, gluten intake activates gluten-specific CD4+ T cells in the lamina propria, upregulating IL-15, a proinflammatory cytokine (Sanz, 2015). Moreover, gut microbiota can also activate Th1-, Th2-, and Th17-mediated immune responses similar to upregulation by gliadin peptide (Sjöberg et al., 2013).

Finally, dysbiosis may also increase the amount and size of gliadin peptides due to differential peptidolytic activity of the gut microbiota (Herrán et al., 2017; Kõiv and Tenson, 2021). Recent research has shown that peptidases from different sources can degrade gluten and gluten-derived peptides (Herrán et al., 2017; Kõiv and Tenson, 2021). In this regard, several bacteria from the human digestive tract (i.e., *Bifidobacterium* spp., *Lactobacillus* spp., and *Rothia* spp.) can potentially degrade gluten, and a healthy microbiome composition could modulate the symptoms of gluten-related diseases (Herrán et al., 2017). These studies suggest that the gut microbiota affects gluten digestion, intestinal permeability, and the host immune system, all the mechanisms involved in the pathogenesis of CeD.

Strict adherence to a Gluten-Free Diet (GFD) and a lifelong exclusion of gluten from the diet is the first-line treatment and is currently the only effective therapy for CeD (Al-Toma et al., 2019). GFD has become a trend in contemporary history, being associated with increased energy and health; however, evidence suggests that GFD is an unbalanced diet with multiple nutritional deficiencies (Lerner et al., 2019). People undertaking gluten-restricted products are often associated with diets containing an inadequate nutritional value, characterized by a higher fat intake, less vegetable-protein intake, and higher carbohydrate and sugar consumption (Lerner et al., 2019; Melini and Melini, 2019). Although GFD can reduce the symptoms of CeD in most patients, it does not entirely restore the gut microbiota to that of healthy individuals; moreover, it has been reported that up to 30% of patients will exhibit non-responsive CeD, a condition characterized by the persistent enteropathy and CeD-related symptoms after 1 year on a GFD (Leonard et al., 2017).

Despite the evidence suggesting the implication of gut microbiome over CeD pathogenesis and symptoms persistence after treatment, there is no consensus about the specific microbial changes observed in this pathology. Previous studies have focused on identifying a microbiome composition at an early age in infants that could be predictive of CeD development (Sellitto et al., 2012; Olivares et al., 2018; Rintala et al., 2018), the effect of the time of first gluten exposure (Sellitto et al., 2012), and other environmental factors such as delivery mode, antibiotic exposure, and infant-feeding type on microbial gut composition and/or CeD development (Leonard et al., 2020). Additionally, researchers discovered that several microbial species, pathways, and metabolites are altered in abundance in infants at high risk of developing CeD before the disease manifests, suggesting that HLA-DQ alleles can affect early gut microbiota composition (Leonard et al., 2021), pointing out the influence of host genetics over the gut microbiome. Other studies in adults comparing healthy patients vs. patients with CeD have demonstrated that alpha-diversity between samples from patients with CeD and other groups did not show differences. However, specific changes in taxa abundance were found, for example, the increase of *Proteobacteria* in patients with CeD and a decrease in *Firmicutes* and *Actinobacteria*, evidencing the existence of a dysbiosis in CeD with a predominance of Gram-negative bacteria (Cheng et al., 2013; Pellegrini et al., 2017; Bodkhe et al., 2019; Panelli et al., 2020).

Differences were observed across studies regarding the use of different sequencing technologies, experimental approaches and analysis pipelines, difficult cross-studies comparison, and microbial markers’ establishment to evaluate disease progression. Here we performed a comprehensive study of the microbiome in CeD, combining multiple datasets of 16S rRNA gene sequencing available in public databases. We compiled datasets from parts of the gastrointestinal tract (GIT) and extensive metadata, considering the influence of GFD over the gut microbiome, trying to find microbial biomarkers for CeD not only in the duodenum but also in less invasive samples such as saliva, stool, and oropharynx exudates. Finally, we developed for the first time a model to classify patients with CeD based on their gut microbiota composition.

## Results

We found nine out of the nineteen selected studies meeting the inclusion criteria as shown in Supplementary Table 1 for the merged data analysis (Table 1). Table 2 shows the number of samples for each study, the clinical classification, and tissue of origin (stool, duodenum, pharynx, or saliva) for the data included in the analysis. Finally, we included 435 total samples, comprising 190 patients with active or treated CeD and 245 controls.

**TABLE 1:** The number of samples for each study, clinical classification, and tissue of origin for the data included in the analysis.

References	Accession N° ENA^a^	Sampled tissue	Sequencing technology	16S region
Bodkhe et al., 2019	PRJNA385740	Duodenum and stool	Illumina Miseq	V4
Garcia-Mazcorro et al., 2018a	PRJNA401920	Duodenum and stool	Illumina Miseq	V4
Iaffaldano et al., 2018	PRJNA371697	Pharynx	Illumina Miseq	V4-V6
Olivares et al., 2018	PRJEB23313	Stool	Illumina Miseq	V1-V2
Tian et al., 2017	PRJNA321349	Saliva	Illumina Miseq	V3-V4
Bonder et al., 2016	PRJEB13219	Stool	454	V3-V4
Giacomin et al., 2016	PRJNA316208	Duodenum	454	V1-V3
Quagliariello et al., 2016	PRJEB14943	Stool	Illumina Miseq	V3-V4
Francavilla et al., 2014	PRJNA231837	Saliva	454	V1-V3

The cases (*n* = 190) include patients with celiac disease, on a diet with or without gluten, the controls (*n* = 245) include non-celiac patients with gluten intolerance, gluten-free diet, or without any enteropathy. The total samples add up to 435.

^a^Accession numbers in the “European Nucleotide Archive” (ENA) database.

**TABLE 2:** Statistics of input data to analyze (after filtering samples and ASVs).

Tissue	Feeding habit	ASVs	Samples	Cases	Controls
Duodenum	GFD	475	30	12	18
Duodenum	Unrestricted	475	89	40	49
Stool	GFD	374	89	52	37
Stool	Unrestricted	374	107	24	83
Saliva	Control unrestricted and case GFD	120	78	40	38
Pharynx	Control unrestricted and case GFD	71	42	22	20

ASV, Amplicon Sequence Variant; GFD, Gluten free diet; Cases, Samples from patients with Celiac disease.

We combined 16S RNA sequencing gene datasets for the first time and performed an analysis following the same pipeline. We compared sequences generated from different regions of the 16S rRNA gene by using a reference mapping protocol for amplicon sequence variant (ASV) assignment, in which sequences from different regions of the 16S rRNA gene will map to the same full-length reference sequence from the SILVA SSU v.138 database (Glöckner et al., 2017) if they are from the same species. We could perform an integrative analysis including a high number of samples from different body sites and extensive metadata to find microbial biomarkers characteristic of CeD, taking into account the type of diet.

### Diversity and microbial composition

**FIGURE 1:**
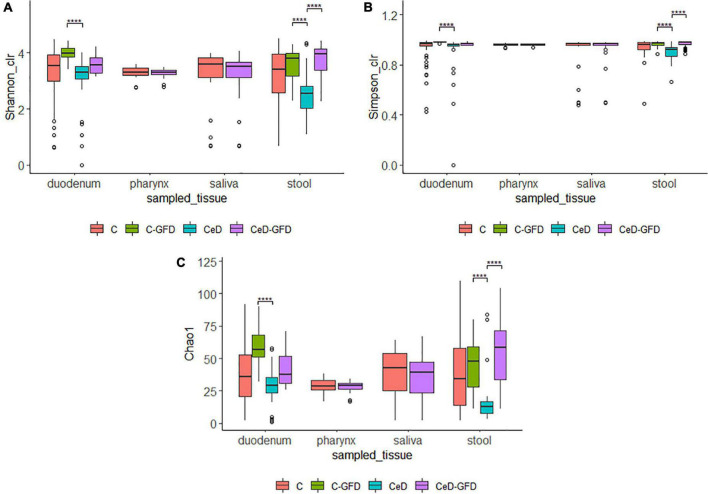
Shannon **(A)**, Simpson **(B)**, and Chao1 **(C)** diversity indices for (1) the pooled samples in cases and controls for each sampled tissue according to diet. The *p*-value was calculated using the Wilcoxon test in **(A,B)** and the Welch’s t-test in **(C)**. The limits of the rectangle indicate the 25th and 75th percentiles and the horizontal bars indicate the median, in **(C)**, the median equals average. Vertical bars indicate upper and lower distribution limits, and dots represent mild outliers. ****P < 0.0001.

Alpha-diversity of the microbiome was estimated using the Chao1, Shannon, and Simpson indices (Figure 1). We did not find differences among healthy controls and patients with CeD regarding alpha-diversity indexes from saliva and pharynx samples. However, when considering duodenum and stool samples, we found an increment in the diversity in patients as in healthy controls undergoing a GFD.

We studied the differences among groups in each tissue sampled by PCoA using weighted UniFrac distance for the beta-diversity analysis (data not shown). We did not find a clear separation when analyzing biological variables (age, type of diet, or clinical condition). Permutational multivariate analysis of variance (PERMANOVA) for each variable by ADONIS function revealed that Study Accession, in the case of the duodenum, saliva, and stool, was a factor influencing the grouping of samples. This may be explained by the experimental protocols used in each study, including differences in the sequencing platform, the region of 16S rRNA targeted, and the DNA extraction technique used, suggesting that some particular protocols may induce some biases.

### Differential analysis of the microbiota, correlation, and biomarker finding

**FIGURE 2:**
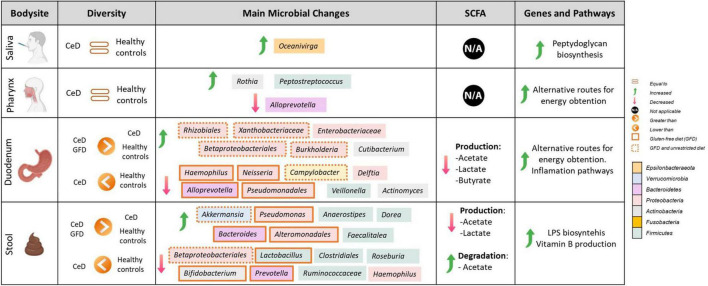
Comprehensive map of microbial and functional changes observed after data integration. SCFA, short-chain fatty acids; CeD, Celiac disease patients. Figure was created with BioRender.com.

To establish microbial biomarkers, first, we conducted a biomarker-finding analysis using the LEfSe tool, followed by a differential abundance analysis using DESeq2 to identify ASVs that were differentially expressed according to studied groups. Finally, a correlation analysis looking for an association between CeD and microbial composition was performed. Figure 2 summarizes the main findings obtained in each tissue analyzed.

#### Microbial changes associated with duodenal microbiota in celiac disease

We found that bacteria of the phylum *Proteobacteria* were characteristic of a CeD patient’s duodenum, with different genera present according to the type of diet.

**FIGURE 3:**
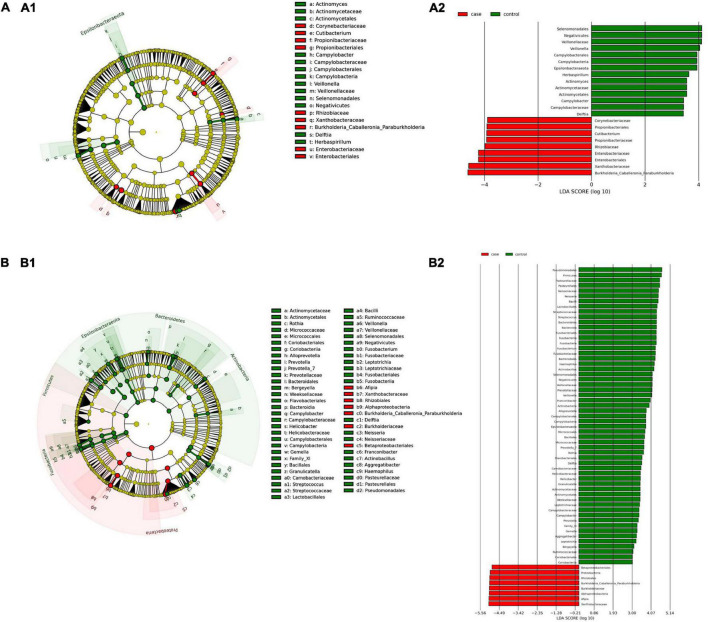
Linear discriminant analysis (LDA) integrated with effect size (LEfSe). Cladogram representing the differentially abundant taxonomic groups in the microbiota from the duodenum of CeD following an unrestricted diet **(A1)** or a GFD **(B1)**. Graph bar representing the microbial biomarkers found in the microbiota from the duodenum of CeD following an unrestricted diet **(A2)** or Patients with CeD following a GFD **(B2)** (LDA score > 3, *p* < 0.001). Control: Control group, represented in green. Case: Patients with CeD, represented in red.

**FIGURE 4:**
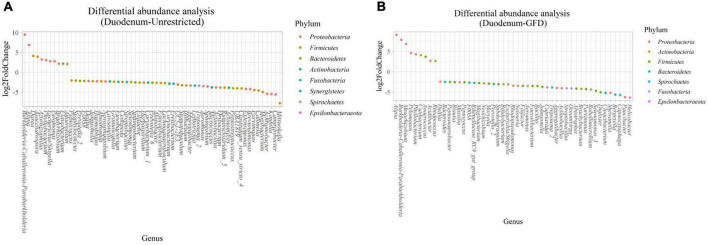
Differential abundance analysis performed on samples from the duodenum of Patients with CeD following an unrestricted diet **(A)** and Patients with CeD under a GFD **(B)** compared with healthy controls.

*(i) Microbial changes of duodenal microbiota from untreated patients with CeD undergoing an unrestricted diet.* For duodenal samples of patients undergoing an unrestricted diet, we found 23 ASVs with an linear discriminant analysis (LDA) score greater than 3 (Figure 3A). Nine ASVs were associated with patients with CeD mainly from *Proteobacteria* phylum, particularly bacteria from the *Burkholderia–Paraburkholderia–Caballeronia* clade, *Alphaproteobacteria*, and *Enterobacteria*, and *Actinobacteria* from the family Corynebacteriaceae. On the other hand, healthy controls were enriched in the classes *Negativicutes* and *Epsilonbacteraeota* of the phylum *Firmicutes.* After differential relative abundance analysis, we found 93 ASVs with significant changes in abundance (FDR < 0.01) (Figure 4A). Among them, 61 were decreased in CeD, and 11 were increased in CeD. Significant ASVs belong to phyla *Firmicutes, Bacteroidetes, Proteobacteria, Epsilonbacteraeota, Actinobacteria, Spirochaetes, Fusobacteria*, and *Synergistetes*. Finally, we found a negative association between CeD and the genus *Actinomyces*, whereas the top five positively associated genera were *Coprococcus* 3, *Hydrocarboniphaga*, *Ruminococcaceae* UCG010, *Cutibacterium*, and *Deinococcus*.

*(ii) Microbial changes of duodenal microbiota from patients with CeD undergoing a GFD.* We selected healthy controls and patients undergoing a GFD to study the microbial composition between the two groups. Biomarker finding analysis revealed 65 ASVs with an LDA score greater than 3 (Figure 3B). Eight ASVs were associated with patients with CeD, mainly from the phylum *Proteobacteria* belonging to the *Burkholderia–Paraburkholderia–Caballeronia* clade, alphaproteobacteria *Afipia*, and order Rhizobiales. On the other hand, 57 ASVs were related with healthy controls following a GFD, particularly from the phyla *Firmicutes*, *Fusobacterium*, *Actinobacteria*, and *Epsilonbacteraeota* comprising the genera *Leptotrichia*, *Fusobacterium*, *Rothia*, and *Campylobacter*, and other *Proteobacteria* (*Neisseriaceae*, *Pseudomonales*, and *Haemophilus*). After performing the differential abundance analysis, we found 49 ASVs with significant changes in abundance (FDR < 0.01) (Figure 4B). Nine were increased in CeD, whereas the other 40 ASVs were decreased. Significant ASVs belong to the phyla *Actinobacteria, Proteobacteria, Firmicutes, Epsilonbacteraeota, Bacteroidetes, Fusobacteria*, and *Spirochaetes.* Finally, the top five negatively associated genera from the duodenum of patients with CeD undergoing GFD were *Haemophilus*, *Neisseria*, *Alloprevotella*, *Fusobacterium*, and *Delftia*. In contrast, the top four positively associated genera were mainly from the phylum *Proteobacteria*, particularly from the order Alfaproteobacteriales, namely, *Falsirhodobacter*, *Asinibacterium*, *Azonexus*, and *Blastomonas*.

#### Microbial changes associated with stool microbiota in celiac disease

**FIGURE 5:**
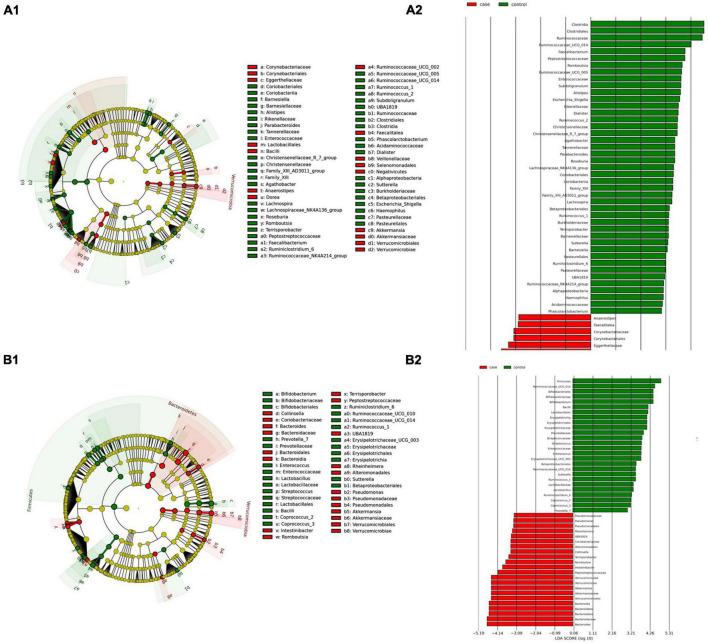
Linear discriminant analysis (LDA) integrated with effect size (LEfSe). Cladogram representing the differentially abundant taxonomic groups in the microbiota from the stool of Patients with CeD following an unrestricted diet **(A.1)** or a GFD **(B.1)**. Graph bar representing the microbial biomarkers found in the microbiota from the stool of Patients with CeD following an unrestricted diet **(A.2)** or Patients with CeD following a GFD **(B.2)** (LDA score > 3, *p* < 0.001). Control: Control group, represented in green. Case: Patients with CeD, represented in red.

**FIGURE 6:**
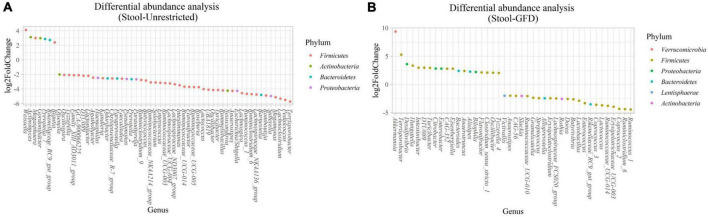
Differential abundance analysis performed on samples from the stool of patients with CeD following an unrestricted diet **(A)** and patients with CeD under a GFD **(B)** compared with healthy controls.

Stool samples are more representative of the colonic microbiota; however, they could also indicate changes related to the disease. Like in duodenum samples, we found an increase in bacteria of the phylum *Proteobacteria*, but different genera were enriched according to the type of diet. Specific changes found were as follows:

*(i) Microbial changes of stool microbiota from untreated patients with CeD undergoing an unrestricted diet*. After biomarker analysis, we found 60 ASVs with an LDA score greater than 3 (Figure 5A). Of them, 17 were associated with CeD, and bacteria from phylum *Verrucomicrobia* and *Firmicutes* were characteristics of this group, mainly the genera *Akkermansia*, *Anaerostipes*, *Faecalibacteria*, and *Dorea*; on the other hand, in healthy controls undergoing an unrestricted diet ASVs from phylum *Proteobacteria* (*Betaproteobacteriales*) and *Firmicutes* mainly order Clostridiales were overrepresented. Differential abundance analysis revealed 74 ASVs belonging to phyla *Firmicutes, Bacteroidetes, Actinobacteria*, and *Proteobacteria* with significant changes in abundance (FDR < 0.01) (Figure 6A). Among them, 56 were decreased in CeD, and seven were increased in CeD. Finally, the top five genera positively associated with CeD were *Achromobacter*, *Flavisolibacter*, *Geodermatophilus*, *Candidatus Rubidus*, and *Tepidimonas*.

*(ii) Microbial changes of stool microbiota from patients with CeD undergoing a GFD*. Biomarker-finding analysis revealed the presence of 48 ASVs with LDA > 3 (Figure 5B). In total, 22 were associated with patients with CeD, mainly from phylum *Verrucomicrobia, Bacteroides, Firmicutes*, and *Proteobacteria*, particularly genera *Akkermansia*, *Bacteroides Romboutsia*, and *Pseudomonas*. On the other hand, 26 ASVs were related to controls undergoing GFD, mainly bacteria from phylum *Firmicutes* and *Actinobacteria* from genus *Lactobacillus*, *Streptococcus*, *Ruminoccocous*, and *Bifidobacterium*. Differential abundance analysis revealed changes in 77 ASVs (FDR < 0.01) (Figure 6B). Among them, 41 were decreased in CeD, and 19 were increased in CeD. Significant ASVs belong to phyla *Firmicutes, Proteobacteria, Bacteroidetes, Verrucomicrobia, Actinobacteria*, and *Lentisphaerae*. Finally, correlation analysis showed 15 ASVs with a significant correlation with CeD. Five were negatively correlated with CeD (*Ruminococcus* 1, *Ruminococcaceae* UCG014, *Ruminiclostridium* 6, *Coprococcus* 2, *Enterococcus*), and ten ASVs have a positive correlation. The top five genera that positively correlated were *Intestinibacter*, *Akkermansia*, *Ruminococcaceae UBA1819*, *Flavonifractor*, and *Terrisporobacter.*

#### Microbial changes associated with pharynx microbiota in celiac disease

**FIGURE 7:**
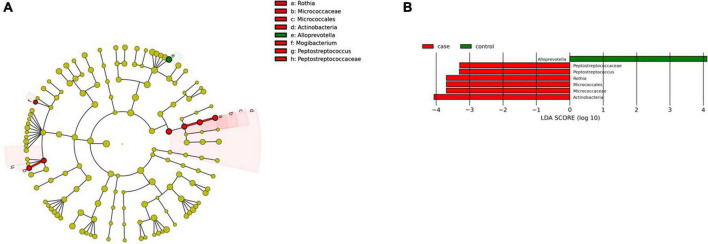
Linear discriminant analysis (LDA) integrated with effect size (LEfSe). **(A)** Cladogram representing the differentially abundant taxonomic groups in the microbiota from the pharynx of patients with CeD. **(B)** Graph bar representing the microbial biomarkers found in the microbiota from the pharynx of patients with CeD (LDA score > 3, *p* < 0.001). Control: Control group, represented in green. Case: Patients with CeD, represented in red.

We found two microbial biomarkers characteristic of the pharynx in patients with CeD: *Rothia*, a nitrate-reducing bacteria usually found in the oral cavity of humans (Rosier et al., 2020), and *Peptosptreptoccus*, an oral pathogen recently associated with Colorectal Cancer development (Ternes et al., 2020; Figure 7A). On the other hand, differential abundance analysis showed significant changes in abundance (FDR < 0.01) of five ASVs. *Veillonella, Mogibacterium, Streptobacillus* (phylum *Fusobacteria*), and *Mannheimia* were increased in CeD, whereas *Treponema 2* was decreased.

*Microbial changes associated with microbiota from saliva samples in CeD.* Despite the most significant changes found in the microbial composition on other parts of the gastrointestinal tract, we only found one genus related to CeD in saliva and was identified after differential abundance analysis and biomarker finding, namely, genus *Oceanivirga* belonging to the phylum *Fusobacteria*, family Leptotrichiacea.

### Prediction of the metabolic functions profiles in bacterial communities

To study the metabolic potential of the microbiota, the analysis was first carried out for all the genes and pathways detected and, since the intestinal microbiota, after its contribution to the digestive process of food, produces short-chain fatty acids that influence the maturation, maintenance, and behavior of the mucosal immune system, we focused on the genes and pathways involved in the production of these compounds. Figure 2 summarizes the main findings observed according to each tissue analyzed.

First, we searched for the 26 genes reported as potentially involved in the biosynthesis pathways of the main SCFAs produced by the human microbiome (butyrate, acetate, and lactate), given their involvement in the modulation of the immune system. Genes included were those identified by Zhao et al. (2019) by gene deletion and overexpression experiments in *E. coli*. These authors identified six genes needed in acetate production (*menI, tesA, yciA, fadM, tesB*, and *ybgC*), eight genes used in butyrate (*entH, tesA, ybgC, ybhC, yicA, menI, yigI*, and *tesB*) production, and two genes required for lactate (*mgsA* and *lldD*) production in addition to the previously known genes *pta-ackA*, *ptb-buk, ldhA, poxB, eutD, tdcD, dld*, and *ykgF* (Zhao et al., 2019). Table 3 shows the number of genes found according to each tissue and the differentially abundant genes/routes between patients with CeD and healthy controls.

**TABLE 3:** Statistics of significant results obtained on Picrust2 predictions analysis.

Tissue	Feeding habit	Gene differential abundance	Gene correlation	Pathway differential abundance	Pathway correlation
Duodenum	GFD	412	0	13	0
Duodenum	Unrestricted	404	0	8	0
Stool	GFD	31	69	0	1
Stool	Unrestricted	261	0	2	0

GFD, Gluten-free diet.

FC = 3, *p* < 0.01.

Differential abundance analysis of metabolic pathways is summarized in Supplementary Table 2. It revealed an increase, in the duodenum of patients with CeD, in the degradation of D-glucarate, L-arabinose, D-galactarate, and biogenic amines, and a decrease in the degradation of lactose and galactose. Regarding genes involved in the production of SCFA, we found a decrease in the abundance of genes involved in the production of acetate (*ackA*) and lactate (*ldhA*) and a negative correlation with genes involved in the production of acetate (*pta*), lactate (*lldD, ldh, dld*, and *tesB*), and butyrate (*tesB*). A decrease in the abundance of the fermentation pathway from hexitol to SCFAs involving the *ackA* and *pta* genes was consistently found.

In stool samples of patients with CeD, the synthesis routes of lipopolysaccharide (LPS) components of the membrane of Gram-negative bacteria, acetate degradation routes, and vitamin B synthesis (B1 and B9) were found to be increased. Regarding genes implied in SCFA synthesis, correlation analysis revealed that two genes related to acetate and lactate production (*pta* and *dld*) were negatively associated with CeD (FDR ≤ 0.01; correlation ≥ 0.2).

No statistically significant differences were found in the abundance of any of the 26 genes related to SCFA production regarding saliva and pharynx samples. However, in saliva samples of patients with CeD, we found an increase in peptidoglycan synthesis and the intermediate degradation routes of aromatic compounds and amino acids.

### Prediction of genes and genes coding for prolyl peptidase enzymes

Some microbiota components can express enzymes different from those produced by humans and promote the digestion of compounds such as the immunogenic peptides of gluten (Herrán et al., 2017; Kõiv and Tenson, 2021). We analyzed four enzymes involved in the degradation of immunogenic gluten peptides that could be involved in reducing CeD symptoms (general N-type aminopeptidase (PepN), X-prolyl dipeptidyl aminopeptidase (PepX), endopeptidase (PepO), and endoprolyl peptidase (PREP). However, none of the four enzymes involved in the degradation of gliadin peptides were found to be differentially abundant between cases and controls or associated with CeD in any of the tissues.

### Use of the random forest machine learning model to discriminate sample groups

To learn about the extent to which the microbial components differed among sample groups of the gastrointestinal tract and the capacity to use microbial information to discriminate patients with CeD, we established random forest (RF) classifiers. The low number of samples from the upper GIT (saliva and pharynx) led us to construct models only with information from the lower GIT (stool and duodenum) to discriminate between healthy controls and patients with CeD. We constructed six models by pooling samples from the duodenum and stool and with/without gluten ingest information. We decoded 16S rRNA hypervariable region information as a binary feature and estimated their effects on our RF models. Additionally, we conducted the same pooling RF analysis without adding 16S rRNA hypervariable region information and achieved a similar performance.

**FIGURE 8:**
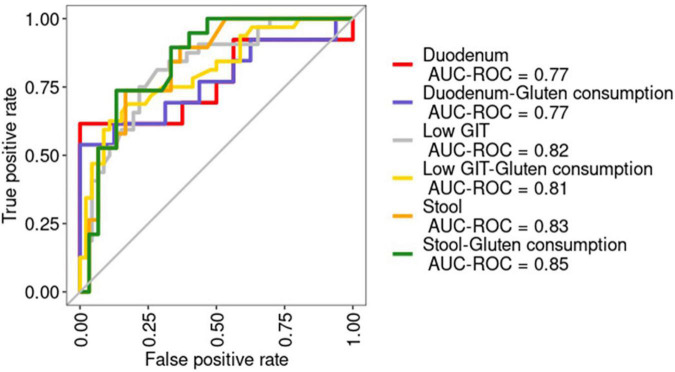
Receiver operating characteristic (ROC) curves for the six models built. The area under the ROC curve (AUC) is shown. AUC-ROC, Area under the ROC curve. Duodenum: RF model using duodenum samples. Duodenum-Gluten consumption: RF model using duodenum samples including information about gluten consumption. Low GIT: RF model using pooled samples from the lower gastrointestinal tract (stool and duodenum). Low GIT—gluten consumption: RF model using pooled samples from the lower gastrointestinal tract (stool and duodenum) including information about gluten consumption. Stool: RF model using stool samples. Stool—gluten consumption: RF model using stool samples including information about gluten consumption.

**FIGURE 9:**
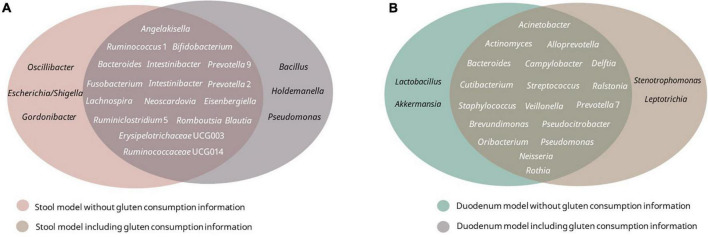
Top 20 best-ranked microbial features of each model. **(A)** Stool model with/without gluten consumption; **(B)** duodenum model with/without gluten consumption.

Receiver operating curves were calculated together with their AUC for each class (Figure 8A). The AUC for the duodenum model was the lowest. In stool and lower GIT models, including gluten ingestion as a feature, was among the top-ranked features, whereas it did not have much importance in the duodenum model. The top-ranked genera in the six models with and without gluten consumption information are shown in Figure 9. The genera *Bifidobacterium, Cutibacterium, Intestinibacter, Oribacterium, Prevotella*, and *Ruminococcaceae* UCG014 were microbial markers shared between models.

Finally, we built a binary RF classifier based only on stool data to evaluate their usefulness as a surrogate marker of the disease using the top-ranked features of the previous stool model (without gluten consumption as a feature). Predictions on the test yielded an AUC of 0.85 (Figure 8B). Afterward, we applied the model to a healthy population for validation obtaining an AUC of 0.75 (Figure 8B).

## Discussion

Our study provides an unprecedented analysis of metataxonomic data from patients with CeD, including (*n* = 435) samples from patients and healthy controls for different body sites. One of the most critical limitations currently found is the lack of standardized methodologies for downstream analyses of sequencing approaches, introducing statistical biases and subsequent challenges for reproducibility and cross-study comparisons (Moreno-Indias et al., 2021). Despite some attempts to standardize methods, a gold standard for microbiome research is not established (Knight et al., 2018). Herein, we create an extensive dataset processed following the same methodology and levering metadata to solve this issue; moreover, we made data publicly available following FAIR principles (Wilkinson et al., 2016).

Celiac disease mainly affects the small intestine (duodenum); according to the European Society for the Study of Coeliac Disease (ESsCD) guidelines, the diagnosis of CeD relies on a combination of clinical, serological, and histopathological findings, and small-bowel biopsy specimens are fundamental for an accurate diagnosis (Al-Toma et al., 2019). In this regard, we studied duodenal microbiota intending to find microbial markers associated with this disease. On the other hand, biopsy-sparing diagnostic guidelines have been proposed and validated in a few recent prospective studies; as the obtention of a duodenal biopsy or duodenal content is an invasive procedure, we aimed to find microbial markers for other parts of the GIT, with less-invasive procedures (stool, oropharyngeal exudate, and saliva) to identify possible microbial markers as “surrogate markers” of the duodenum.

Previous studies have shown that *Proteobacteria* was enriched in CeD (Cheng et al., 2013; Pellegrini et al., 2017; Garcia-Mazcorro et al., 2018a; Bodkhe et al., 2019; Panelli et al., 2020). We recognized the same finding, but we were also able to discern differences in the abundance of particular *Proteobacteria* families according to the type of diet of the patients. For patients with untreated CeD, *Burkholderiaceae*, *Xantobacteriaceae*, and *Enterobacteriaceae* were enriched, whereas in patients under a GFD, we found a decrease in some *Enterobacteriaceae* (duodenum) and *Betaproteobacteriales* (stool). These findings suggest that the lower GIT microbiota from patients with CeD is enriched in *Proteobacteria* with potential implications for activation of immune response and inflammation. In this sense, predicted metabolic pathways found in duodenum and stool samples were characterized on the one hand by the increase in alternative routes for obtaining energy (degradation of D-glucarate, L-arabinose, D-galactarate, and biogenic amines) and a decrease in the production of SCFA. Because the cells involved in the immune response require a large amount of energy for their activation, proliferation, and recruitment of other cells during inflammation, the availability of carbohydrates is scarce for bacteria to synthesize their components. Therefore, they obtain energy by alternative routes, for example, from acetate through the glyoxalate cycle, or obtaining energy are compounds such as D-glucarate, D-galactarate, and biogenic amines (Chauhan and Saha, 2018).

On the other hand, we found a generalized low presence of genes related to the production of beneficial SCFA, with anti-inflammatory potential. The reduction of these compounds is associated with a reduction in the bacterial genus that produces them, such as *Ruminoccous*, *Veillonella*, and *Clostridiales* (as observed in taxonomic analysis) and potentially with a lower fiber intake typical of a gluten-free diet (Lerner et al., 2019). A low abundance of SCFA has been previously reported in CeD at an early stage (Cagno et al., 2011) and may be involved in the predisposition to the development of CeD (Verdu et al., 2015). Moreover, stool samples were characterized by an increase in the synthesis routes of LPS, and vitamin B synthesis (B1 and B9), which is consistent with our findings in the taxonomic analysis revealing an increase in the abundance of *Proteobacteria*. LPSs are key factors in the activation of the immune response through TLR4 signaling, contributing to inflammation, and loss of intestinal permeability seen in CeD (Levy et al., 2017; Salguero et al., 2019).

Regarding the upper GIT, first, we studied the composition of the pharynx microbiota of patients with CeD. Anatomically, the pharynx is part of the upper gastrointestinal tract, directly connected with the esophagus, and is conventionally divided into the nasopharynx, oropharynx, and hypopharynx. Usually, oropharynx exudate, a sample comprising the part of the throat at the back of the mouth behind the oral cavity, is used for clinical diagnosis of microbial infections (Lieberman et al., 2006), being a valuable method for studying microbiota from the upper intestinal tract with a less-invasive procedure than a biopsy (Lemon et al., 2010; Boutin et al., 2017). We found a particular microbial composition in the pharynx of patients with CeD, mainly associated with proinflammatory bacteria (i.e., *Mogibacterium*) and opportunistic pathogens (i.e., *Peptostreptococcus* and *Streptobacillus*). Notably, we found *Rothia* spp., a species containing gluten-degrading enzymes (Kõiv and Tenson, 2021), in non-symptomatic patients undergoing GFD; further studies will be helpful to discriminate the role of this bacteria in CeD and its possible use for monitoring the disease.

We found the genus *Oceanivirga* as a marker of patients with CeD for the first time regarding saliva samples. These bacteria are from the family Leptotrichiaceae, relatively poorly studied Gram-negative bacteria, facultative or obligate anaerobes, found as colonizers of mucous membranes in the oral cavity of humans and other animals (Eisenberg et al., 2018). Particularly, the genus *Oceanivirga* has been isolated in subgingival samples from patients with periodontitis (Hagenfeld et al., 2018), however, validation as a microbial marker of CeD needs to be performed.

Predicted metabolic pathways analysis revealed an increase in the intermediate degradation routes of aromatic compounds and amino acids in the pharynx samples, both routes associated with energy acquisition from bacteria (Chauhan and Saha, 2018) and an increase in peptidoglycan synthesis in saliva. Peptidoglycans are part of the cellular wall of bacteria and, besides LPS, are responsible for microbial inflammation mediated by the innate immune response (Amoureux et al., 2005).

Finally, we developed a novel RF classifier for discerning between patients with CeD and healthy controls. We used an RF algorithm in view of its usefulness when applied to microbiome datasets (Marcos-Zambrano et al., 2021; Moreno-Indias et al., 2021). Due to the small sample size for upper GIT samples, we could only build RF models for lower GIT. Some of the microbial markers identified by LEfSE, differential abundance expression, and correlation in stool and duodenum samples were also present in the RF models (*Alloprevotella spp., Cutibacterium* spp*., Delftia* spp*., Neisseria* spp., and Rothia spp.), demonstrating their potential as microbial biomarkers able to discern between the disease and its possible usefulness in combination with other prediction models to estimate the risk of having CeD based on symptoms and risk factors previously described (Elwenspoek et al., 2022). The RF model built with stool samples achieves an outstanding performance even with the validation dataset proving the capability of using a stool as a surrogate marker for changes in the duodenal microbiota of patients with CeD.

One of the main limitations of our study is the low number of samples from patients with CeD included, and we were restricted by the datasets available and the scarce metadata, making open access data mandatory not only for reproducibility but also for increasing the number of available resources and increasing the body of information about specific diseases. On the other hand, we could only provide information about microbial markers at the genus level; the use of whole-genome sequencing data will be beneficial to discern specific strains implicated in the changes evidenced in our study.

However, despite limitations, we were able to find microbial markers related to CeD at different body sites, including SCFA production and the prevalence of inflammatory pathways (Figure 2). Our results showed coordinated changes throughout the GIT, with specific changes according to each body site. In the upper GIT, we found an enrichment in *Actinobacteria* from the genus *Rothia* in the pharynx, and *Cutibacterium* in the duodenum, and a marked decrease in *Alloprevotella* spp. in both sites. Whereas, the lower GIT presents more changes characterized by an increase in *Proteobacteria* and a decrease in *Actinobacteria*, *Campylobacter*, and SCFA producers such as *Ruminococaceae*, and *Clostridiales*. Moreover, we define some differences in gut microbiota (from duodenum and stool samples) between untreated patients with CeD following an unrestricted diet and patients with CeD following GFD. Although we could not find restoration in microbial dysbiosis, we found that patients following a GFD have an overall higher microbial diversity and an abundance of certain bacteria usually related to health benefits such as *Akkermasia* (Derrien et al., 2017) and a decrease in the presence of *Enterobacteriaceae*.

Future prospective studies will provide the “solution” to which comes first, the chicken or the egg? That is, dysbiosis led to the disease, or does the disease produce dysbiosis? Current studies have a cross-sectional design and perform descriptive association at a snapshot of time; CeD is the perfect scenario for studying the implications of microbiota over the pathophysiology of a disease, the trigger of the pathology (i.e., gluten) is traceable, and the genetic environment predisposing to the disease is also knowledgeable.

Celiac disease diagnosis is challenging because the symptoms are varied and non-specific. Most people with CeD remain undiagnosed, and it takes an average of 12 years to get the correct diagnosis (West et al., 2019). Active case findings can help combat underdiagnosis by offering CeD tests to people at higher risk of CeD. In this sense, using prediction models to estimate the risk of having CeD based on symptoms and risk factors is helpful; however, the performance of these models is lower when using only clinical data (Elwenspoek et al., 2022). The use of microbial markers isolated from stool samples, besides being a non-invasive procedure, will help to improve the predictive power of current models. On the other hand, integrating clinical markers of mucosal integrity and microbial markers could be a proper tool for follow-up of the disease. Previous research has shown that the gut microbiota of subgroups of patients with CeD with different clinical manifestations varies, suggesting that gut microbiota play a role in the persistence of symptoms even after adherence to a GFD (Garcia-Mazcorro et al., 2018b). A combination of clinical models and microbial markers—as identified in this research—for diagnosis and follow-up of the disease would substantially improve current clinical practice.

Finally, there is a need to generate larger datasets and properly apply machine learning (ML) that would help to generate helpful and more universally applicable results. Moreover, evaluating the usefulness of stool, oropharyngeal exudates, and saliva as surrogate markers of the microbial state of the duodenum for the diagnosis and management of patients with CeD would be necessary. Last but not least, additional research is required to determine the potential efficacy of gut microbiota modulators such as probiotics and prebiotics as adjuvant treatments for CeD based on the microbial biomarkers linked to symptom persistence and disease pathogenesis.

## Materials and methods

### Literature search, identification, and selection of relevant studies

We conducted a systematic and comprehensive literature search of the studies from 2010 to August 2020, which carried out metataxonomic sequencing of the 16S rRNA gene for microbiomes of patients with CeD, including those on a GFD as well as on an unrestricted diet. We searched on PubMed, Google Scholar, and SCOPUS. The terms used for the search, the automatic filters, and the statistical results are summarized in Supplementary Table 1; Supplementary Figure 1 summarizes the methodology used for the scoping review. The studies retrieved with the previous strategy undergo manual curation to exclude non–CeD-related studies and studies using non–high-throughput 16S rRNA gene sequencing, which escaped the automatic filters.

Of the total selected studies, those included in the merged-data analysis met the following criteria: (a) Bacterial 16S rRNA gene sequenced from total DNA using high-throughput sequencing, (b) data must be available in fastq format in one of the publicly accessible databases [NCBI Sequence Read Archive (SRA) or EBI European Nucleotide Archive (ENA)], and (c) metadata on the sequenced biological samples must be available and must include tissue of origin, information on the type of diet (with or without gluten), and clinical classification (case or control).

### Amplicon sequence variant detection and taxonomic assignment

All data from the selected studies were available in the European Nucleotide Archive (ENA) database. Raw 16S rRNA gene sequencing data sets from each study were downloaded from that database. The quality of the sequencing was examined using FastQC v.11.9 software (Andrews, 2010), and the primers used in the PCR amplification of hypervariable regions were removed using the Cutadapt v.2.9 software (Martin, 2011). For data obtained by paired-end sequencing, each pair of reads was joined by overlapping assembling using the software FLASH v.1.2.11 (Magoè and Salzberg, 2011); then, each study was processed individually for the construction of the ASV count table for each sample, using the software DADA2 v.1.15 (Callahan et al., 2016). Briefly, DADA2 was used for quality filtering of the sequences, detecting exact ASVs, removing chimeras, and finally, the taxonomic assignment of ASVs with SILVA SSU v.138 database (Glöckner et al., 2017). The computer code was developed with the R (R Core Team, 2013), and “bash shell” programming environments for GNU/Linux. All the workflow and the specific criteria for each step in the analysis for each dataset are available on GitHub.^1^

### Data merging, filtering, and normalization

Two tables were obtained per study as a result of the variant detection process. The first one with the abundance of each variant (ASV-count table) and the second one, with the taxonomic assignment of ASVs in six taxonomy ranks (Kingdom, Phylum, Class, Order, Family, and Genus). Tables were combined using the *phyloseq_merge* command. With Phyloseq (McMurdie and Holmes, 2013) *taxa_glom* command, taxa were agglomerated at the genus level to avoid ASV duplication bias. All tables, along with raw and processed data and metadata from this study, are available in FigShare.^2^

### Statistical analysis

All the tests were carried out using the Phyloseq (McMurdie and Holmes, 2013) and Vegan v.2.5.6 (Oksanen et al., 2007) packages of the R programming environment. For the representation of statistical significance in the graphs, the following equivalences were used: *p* < “***” - > 0.001, “**” - > 0.01, “*” - > 0.05, “.” 0.1, 1. The bar graphs and the scatter graphs were obtained in the R programming environment using the ggplot2 package (Villanueva and Chen, 2019).

### Estimation of the biological diversity and composition of the microbiome

The Phyloseq v.1.3 package (McMurdie and Holmes, 2013) from R v.3.6.3 (R Core Team, 2013) was used to estimate rarefaction curves for each sample. Rarefaction curves were proper to determine the minimum sequencing depth for reaching the saturation of observed species.

The diversity and richness of each sample were estimated by Shannon, Simpson, and Chao1 indexes using the phyloseq package. The Shannon and Simpson indexes were estimated after normalizing the data using the Centered log-ratio method in the Compositions package (van den Boogaart and Tolosana-Delgado, 2008), while the Chao1 index was estimated on the raw data. The normality of the data was evaluated using the Shapiro–Wilk test. The Wilcoxon–Mann–Whitney test was used to compare diversity means between two groups and the Kruskal–Wallis test for more than two groups. The homoscedasticity of the variances was calculated using Levene’s test. Since the Shannon index data did not meet the criteria of homoscedasticity or normality, the data were adjusted to a normal distribution by square root transformation and subsequently analyzed with the Welch test.

Also, a principal coordinate analysis (PCoA) was performed on weighted Unifrac distances to display whether the data were grouped by any of the variables included in the metadata (case/control and tissue sampled, 16S rRNA gene region, age, gluten/gluten-free diet, and sequencing technology). Before PCoA data analysis, taxa with less than five counts in raw ASV-count tables or being present in just one sample were discarded.

### Differential abundance, association, and linear discriminant analysis for the discovery of microbial markers on celiac disease

Data for each tissue was subsetted from raw ASV-count tables and both data rarefaction and the Total Sum Scaling (TSS) normalization were performed. The taxa present in less than 10% of samples or having less than ten counts were discarded. The CLR transformation was performed on raw ASV-count tables before differential abundance analysis with RNAseq methods, that is, DeSEq2 (Love et al., 2014) and Correlation Analysis.

For the Linear Discriminant Analysis of Effect Size (LEfSe) analysis, LEfSe Conda version 1.0.0 (Segata et al., 2011) was used with an alpha cutoff of 0.05 for feature significance and an effect size cutoff of 3. The difference in taxa abundances between cases and controls for each sampled tissue was evaluated with DESeq2 software v.1.26.0 (Love et al., 2014). Differences with adjusted *p* < 0.01 and Fold change < 3 were considered significant. Association among the taxa at the genus level and CeD were studied by Spearman-rank correlation analysis.

### Inference of the microbiome metabolic potential

The software PICRUSt2 (Douglas et al., 2020), which infers the genes encoded in studying the taxa genomes, was used to predict the microbial communities’ functional and metabolic capacities present in each sample. The results consist of tables of genes, metabolic pathways, and enzyme abundance in each sample. Data were normalized by using the CLR transformation. The differential abundance of metabolic pathways and genes between cases and controls was estimated using DESeq2 software v.1.26.0 (Love et al., 2014). Subsequently, a targeted search for differentially abundant genes whose impact on the CeD pathogenesis is of interest was carried out.

### Random forest

Relative abundances were first filtered to remove markers with low overall abundance and no variance, log-transformed (after adding a pseudo-count of 1E-05), and finally standardized as *z*-scores. Data were split into training and test sets with 75:25 proportion. Random Forest models were built using the caret R package (version 6.0.90) (Kuhn, 2008). Six different models were trained: pooling stool and duodenum samples (lower GIT), stool, and duodenum, including gluten ingest information and without gluten ingest information. 16S rRNA hypervariable region information was decoded as a binary feature. Models were trained by 10 times repeated 10-fold cross-validation (balancing class proportions across folds) while performing a grid search for the mtry hyperparameter and ntree = 500. The impurity decrease at each split was calculated *via* the Gini index criterion. The optimal combination of hyperparameters was chosen based on the model’s accuracy.

Finally, we predicted labels on the test data using the six chosen models, we plotted their receiver operating characteristic curves (ROC) and calculated the area under the curve (AUC) as a quality measure. ROC curves and their corresponding AUCs were calculated using the MLeval package in R (version 0.3). Finally, feature importance was estimated with the varImp function from caret, all features were included to obtain the importance of each feature, from which they were sorted.

### Model validation and biomarker identification

The stool model based exclusively on metagenomic data was chosen for validation and biomarker search. We used the top 20-ranked features from the stool model with the hyperparameters fixed to mtry = 3 and ntree = 500. This model was also used to predict metabolic status in an unpublished cohort of 62 healthy patients, composed of *n* = 60 healthy controls and *n* = 2 patients with CeD.

## Data availability statement

Publicly available datasets were analyzed in this study. This data can be found here: https://figshare.com/projects/An_lisis_del_microbioma_en_Enfermedad_Cel_aca/82547.

## Author contributions

LM-Z and EC: conceptualization and funding acquisition. JA-G and LM-Z: data curation, methodology, formal analysis, and writing—original draft preparation. VL-K and AR: resources. EC, VL-K, and AR: writing—critical review and editing. All authors have read and agreed to the published version of the manuscript.

## Footnotes

1
https://github.com/juearcilaga/Assesing-microbiome-profiles-of-celiac-disease-patients.git

2
https://figshare.com/projects/An_lisis_del_microbioma_en_Enfermedad_Cel_aca/82547
