# Supplementary material for: A comprehensive map of microbial biomarkers along the gastrointestinal tract for celiac disease patients
Source: Front Microbiol. 2022 Sep 13;13:956119. doi: 10.3389/fmicb.2022.956119 (PMC9513315; doi:10.3389/fmicb.2022.956119)
Supplement: Supplementary file 1 [file Data_Sheet_1.docx]

**Figure S1.** Flowchart of the Study selection for datasets used.

**
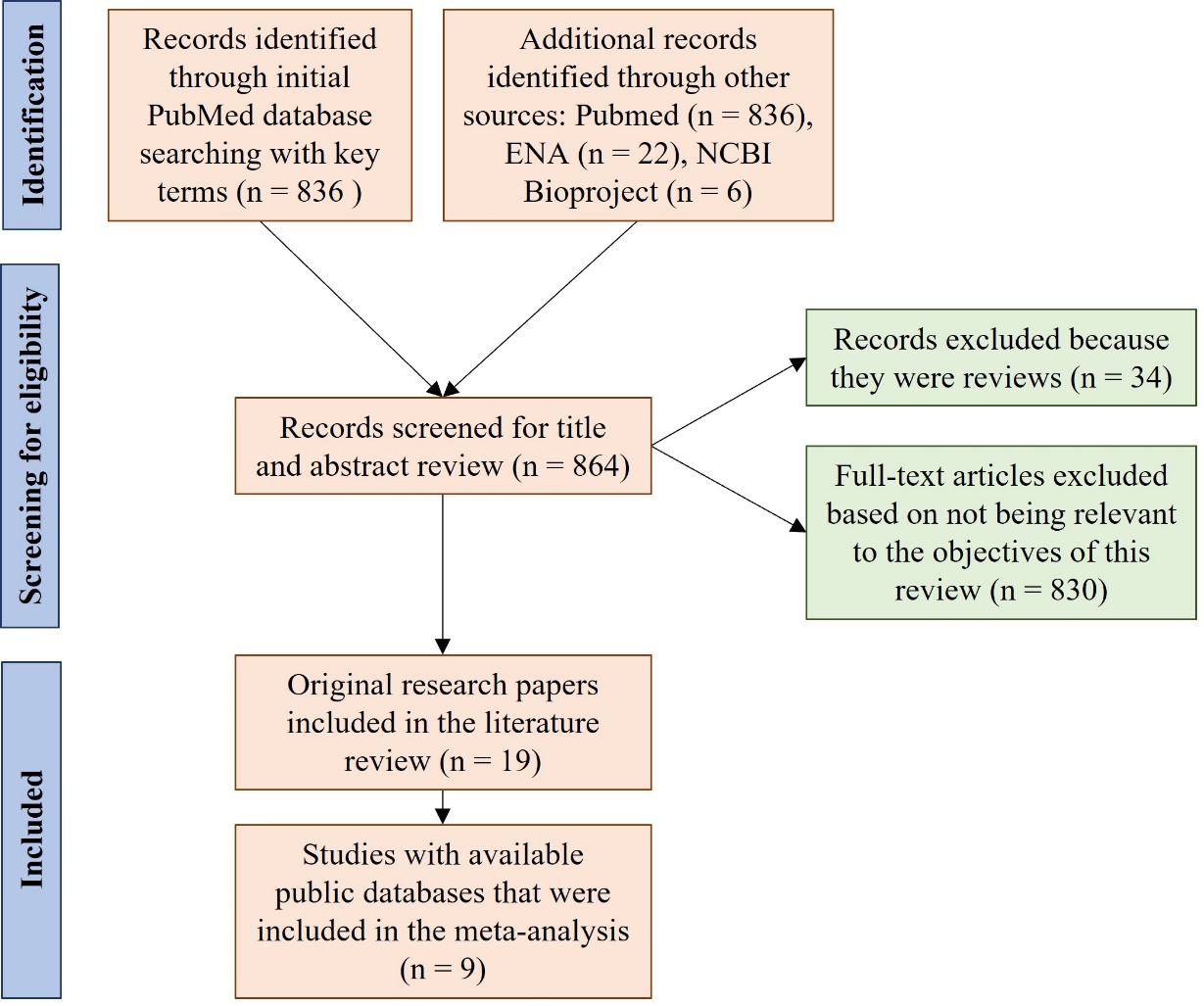
**

**Table S1.** Summary of the criteria used for the literature search and the statistics of the results.

| **Database** | **Target^1^** | **Terms** | **Total** | **New Selected^2^** | **Public data^3^** | **Other filters** |
| --- | --- | --- | --- | --- | --- | --- |
| **Pubmed** | Original research studies with newly generated data | (Celiac OR coeliac) AND (microb*) AND sequencing AND 16S | 21 | 13 | 5 | Full Text, datasets, journal articles, Humans, 2010-2020 |
| **Pubmed** | Original research studies with newly generated data | (Celiac OR coeliac) AND (microb*) AND 16S | 39 | 2 | 0 | Full Text, datasets, journal articles, Humans, 2010-2020 |
| **Pubmed** | Original research studies with newly generated data | (Celiac OR coeliac) AND (microb*) AND metagenomics | 49 | 0 | 0 | Full Text, datasets, journal articles, Humans, 2010-2020 |
| **Pubmed** | Original research studies with newly generated data | (Celiac OR coeliac) AND (microb*) | 727 | 0 | 0 | 2010-2020 |
| **ENA** | Original research studies with newly generated data | microb* AND (Celiac OR Coeliac) | 22 | 4 | 8 | NA |
| **NCBI Bioproject** | Original research studies with newly generated data | microb* AND (Celiac OR Coeliac) AND 16S | 6 | 0 | 5 | NA |
| **Pubmed** | Reviews | (microb*[Title]) AND (celiac[Title] OR coelic[Title]) | 17 | 17 | 0 | Full text, Review, Systematic Review, 2010-2020 |
| **Pubmed** | Reviews | (microb*) AND (Celiac OR coeliac) AND 16S AND Review | 1 | 1 | 0 | Full text, Review, Systematic Review, 2010-2020 |
| **Pubmed** | Reviews | (Celiac OR coeliac) AND (microb*) | 727 | 16 | 0 | 2010-2020 |

^1^ The type of paper intended to find. ^2^The additional studies found with certain terms, which were not found in the previous searches. ^3^The number of studies with data sets available in public databases.

**Table S2.** Results of the analysis of differential abundance of metabolic pathways. Only the differentially abundant pathways are shown in a statistically significant way. Positive values represent an increase in gene abundance in controls compared to cases and negative values represent an increase in gene abundance in cases compared to controls.

| **Pathway** | **Tissue** | **log_2_FC*** | ***P* Value** | **FDR**** | **Controls** |
| --- | --- | --- | --- | --- | --- |
| Degradation of D-glucarate I | duodenum | -4,03 | 3,20E-15 | 1,03E-12 | Disminished |
| Degradation of L-arabinose IV | duodenum | -3,93 | 5,12E-10 | 2,75E-08 | Disminished |
| Degradation of D-glucarate and D- galactarate | duodenum | -3,89 | 1,47E-14 | 1,58E-12 | Disminished |
| Degradation D-galactarate I | duodenum | -3,84 | 1,47E-14 | 1,58E-12 | Disminished |
| Degradation of biogenic amines | duodenum | -3,22 | 2,94E-11 | 2,38E-09 | Disminished |
| Degradation of lactose and galactose I | duodenum | 3,04 | 3,02E-09 | 1,08E-07 | Disminished |
| Fermentation of hexitol to lactate, formate, ethanol and acetate | duodenum | 3,29 | 4,36E-11 | 2,81E-09 | Disminished |
| Biosynthesis of ADP-L-glycero-β-D-manno-heptose | stool | -5,4039 | 6,60E-27 | 1,83E-24 | Disminished |
| Fermentation of acetyl-CoA to butanoate II | stool | -4,6659 | 1,36E-18 | 2,15E-17 | Disminished |
| Thiamine diphosphate II biosynthesis | stool | -4,4658 | 2,89E-22 | 7,69E-21 | Disminished |
| Biosynthesis of the thiazole component of thiamine diphosphate | stool | -4,3899 | 1,39E-19 | 2,75E-18 | Disminished |
| (KDO) 2-lipid IVA transferase III (Chlamydia) | stool | -3,5326 | 5,35E-23 | 2,12E-21 | Disminished |
| CMP-3-deoxy-D-manno-octulosonate biosynthesis | stool | -3,4348 | 1,11E-22 | 3,86E-21 | Disminished |
| Biosynthesis of lipid IVA | stool | -3,4288 | 3,04E-22 | 7,69E-21 | Disminished |
| Guanocine III nucleotide degradation | stool | -3,4273 | 3,28E-18 | 4,79E-17 | Disminished |
| Thiamine diphosphate I biosynthesis | stool | -3,3263 | 1,68E-24 | 1,56E-22 | Disminished |
| Biosynthesis of preQ0 | stool | -3,2554 | 7,14E-25 | 9,92E-23 | Disminished |
| Gluconeogenesis I | stool | -3,1878 | 1,91E-23 | 1,06E-21 | Disminished |
| Nitrate VI reduction (assimilation) | stool | -3,1798 | 1,91E-23 | 1,06E-21 | Disminished |
| Tetrahydrofolate biosynthesis | stool | -3,0552 | 3,68E-14 | 3,79E-13 | Disminished |
| Queuosin biosynthesis (de novo) | stool | -3,0297 | 1,25E-23 | 8,68E-22 | Disminished |
| Synthesis of peptidoglycan II | saliva | -5,4509 | 3,53E-08 | 9,86E-06 | Disminished |
| Degradation of protocatechuate II | pharynx | -9,2733 | 2,40E-09 | 2,85E-07 | Increased |
| Degradation of aromatic compounds by β-ketoadipate | pharynx | 8,6482 | 4,08E-09 | 2,85E-07 | Increased |
| Catechol III degradation | pharynx | 8,6482 | 4,12E-09 | 2,85E-07 | Increased |
| superpathway of 2,3-butanediol I biosynthesis | pharynx | 8,5633 | 4,56E-09 | 2,85E-07 | Increased |
| Catechol degradation to β-ketoadipate | pharynx | 8,4808 | 4,92E-09 | 2,85E-07 | Increased |
| Degradation of L-leucine I | pharynx | 8,3194 | 1,05E-08 | 5,04E-07 | Increased |
| superpathway of 2,3-butanediol II biosynthesis | pharynx | 9,2733 | 2,40E-09 | 2,85E-07 | Increased |
| Catechol degradation to β-ketoadipate | pharynx | 8,6482 | 4,08E-09 | 2,85E-07 | Increased |

* log2 of the Fold Change (FC); that is, log2 of the ratio between the relative abundance of a taxon between cases and controls.

** False discovery rate or p-value adjusted for multiple comparisons using the Benjamini-Hochberg test
